# EGF receptor and COX-1/COX-2 enzyme proteins as related to corresponding mRNAs in human per-operative biopsies of colorectal cancer

**DOI:** 10.1186/1471-2407-13-511

**Published:** 2013-10-30

**Authors:** Annika Gustafsson Asting, Ava Farivar, Britt-Marie Iresjö, Helena Svensson, Bengt Gustavsson, Kent Lundholm

**Affiliations:** 1 Department of Surgery, Institute of Clinical Sciences, Sahlgrenska Academy, University of Gothenburg, Bruna Stråket 20, Gothenburg 413 45, Sweden

**Keywords:** COX-2, EGFR, Colorectal cancer, mRNA, Western blot

## Abstract

**Background:**

Cyclooxygenase (COX) and epidermal growth factor receptor (EGFR) activities promote progression of colorectal cancer. Combined treatment against these targets has not been more effective than single treatments alone. Therefore, our aim was to analyze relationships between COX and EGFR in peroperative colorectal tumor biopsies.

**Method:**

Tumor and colon mucosa tissue were collected at primary intended curative operations in patients according to well-recognized statistical distributions of tumor stages in colorectal cancer. COX-1, COX-2 and EGFR content in tumor and colon mucosa tissue were quantified by western blot and Q-PCR.

**Results:**

COX-2 protein appeared as two bands, one at 66 kDa in almost all tumor and mucosa samples and one at 74 kDa in 73% of the tumors and in 23% of the mucosa samples. Tumor COX-2 mRNA was not different from the content in mucosa samples, while COX-2 protein was increased in tumor tissue (p < 0.0003). A correlation between 74 kDa COX-2 protein and COX-2 mRNA occurred in tumor tissue, with significantly increasing COX-2 mRNA across tumor stages. EGFR mRNA content was lower in tumor tissue (p < 0.0001), while EGFR protein was similar in tumor and mucosa samples. COX-2 and EGFR proteins showed a positive correlation in mucosa, while a negative correlation occurred in tumor tissue. Tumor tissue with high COX-2 74 kDa protein lacked EGFR protein.

**Conclusion:**

Our present results are compatible with the theory that COX-2 and EGFR signalling pathways are inversely related in colorectal cancer tissue. This may explain why combinatorial clinical treatments have been less rewarding.

## Background

Several studies have reported and concluded that inhibition of COX may decrease the risk for colorectal cancer (CRC) and subsequent death [1-5], while other studies have indicated favorable anti-EGFR treatment of CRC; a treatment which is already in clinical use [6,7]. COX and EGFR are thus suggestive targets for combinatorial treatment of CRC since both pathways involve tumor progression and are reported to show increased activities in CRC tissue. There are also published information that increased COX-2 expression may lead to subsequently increased EGFR expression including cross-talk between the two signalling pathways [8]. Accordingly, combined treatment against COX-2 and EGFR activities has been rewarding in animal studies [9]. However, clinical trials with dual blockade of EGFR and COX-2 in patients with CRC have so far not improved clinical outcome compared to the use of the single treatments alone [10].

Previous analyses in our laboratory indicated that tumors treated with COX inhibitors, showed reduction in COX-2 mRNA in combination with decreased expression of EGFR in experimental tumors on mice [11]. However, in a previous study we found unexpected discrepancies between mRNA and protein content of COX-2 in preoperative colorectal tumor biopsies from patients [12]. Most reported studies, mainly based on immunohistochemical analysis of protein content, describe significantly increased expression of COX-2 in tumor tissues while transcript analyses have not confirmed elevated mRNA content of COX-2 in CRC tumor tissue [4,13,14]. It is therefore important to evaluate the relationships between mRNA and protein content of COX and EGFR in human CRC. Therefore, the aim of the present study was to analyze transcripts of COX-1, COX-2, and EGFR in relationship to corresponding proteins in human CRC tissue as well as in normal mucosa tissue.

## Methods

### Patients

Tumor and colon tissue samples from 30 patients were selected to represent a frequently recognized statistical distribution of tumor stages from a large biobank consisting of over 2000 patients who underwent primary operation for colon carcinoma between 2002 to 2009 at Östra Hospital, Sahlgrenska University Hospital Gothenburg Sweden. All patients underwent surgery as the only curative treatment and none had received neoadjuvant radio-chemotherapy, according to individual decisions and institutional indications. The group of patients consisted of 53% males and 47% females with a mean age of 76.9 years (range 51 to 93 years) at surgery. Median survival time was 73.6 months (range 1.7 to 108.5 months) following surgery according to a recent update of survival (Nov 2010), where 13 patients were still alive. Tumors were histologically classified as stadium I (5), II (10), III (10) and IV (5) corresponding to TNM stages T1N0 (2), T2N0 (3), T2N1 (1), T3N0 (9), T3N1 (6), T3N2 (6), T4N0 (1), T4N1 (1) and T4N2 (1). Differentiation of the tumors were 3% high (1), 70% medium (21), and 27% low (8). This study was approved by the board of Ethics at University of Gothenburg (365-05). Accordingly, all patients participated with informed consent.

### Tumor and mucosa tissue material

Tumor and mucosa tissue samples were collected down to the serosa level at primary operation, snap frozen in liquid nitrogen and stored in-80°C until analysis. Certified pathologists staged all tumors. Normal colon tissue was collected at a minimum of 10 cm away from the apparent tumor tissue. Tumor localization was right sided in 73% (22) of the cases and 27% (8) left sided.

### Western blot

50-100 mg of frozen colon mucosa and tumor tissue were thawed and homogenized with a rotor-stator homogenizer in seven volumes of ice-cold RIPA buffer (50 mM Tris pH 7.4, 150 mM NaCL, 0.1% SDS, 1% Nonidet® P-40 Substitute (Igepal™ CA-630), 0.5% Deoxycholic acid) with addition of Complete protease inhibitor cocktail (Roche Diagnostics GmBh, Germany). Homogenates were centrifuged twice at 10 000 × g, 10 min at 4°C and the supernatant was collected. Total protein concentration was determined by the Bradford method using albumin as standard (Quick Start Bradford Protein Assay, Bio-Rad Laboratories Inc.). 30 μg protein from each supernatant were separated in 4-12% NuPage Bis-Tris minigels using Mops buffer system, according to the manufacturer’s instructions (Life technologies), and transferred to PVDF membranes. Membranes were blocked in 10% non-fat dry milk in Tris-buffered saline containing 0.05% tween 20 (NFDM/TBST) for a minimum of two hours. Next, membranes were incubated over night at +4°C with antibodies (rabbit monoclonal anti-EGFR (detects intracellular domain, immunizing peptide aa 1185-1210), Millipore, 04-338; mouse monoclonal anti-EGFR (detects extracellular domain, immunizing peptide aa 30-198) Dako M7298; rabbit polyclonal anti-COX2, Abcam ab15191 is known to detect one distinct band of 72-74 kDa; rabbit monoclonal anti-COX1, Epitomics 3811-1; rabbit polyclonal anti-GAPDH, Millipore Abs-16). Blots were then washed, incubated with HRP-labelled secondary antibodies for 1 hour at room temperature and developed using ECL Prime Western Blotting Kit according to the manufacturer’s description (Amersham Biosciences, UK). Signal was captured using ChemiDoc XRS imaging system (BioRad Laboratories, Sundbyberg, Sweden). After detection of signals, HRP activity was inactivated by 2 × 30 min incubation in hydrogen peroxide buffer (15% H_2_O_2_ in PBS) before reprobing membranes with next antibody. Membranes were cut horizontally after transfer and the lower part were incubated with GAPDH antibodies while the upper part of the membranes were incubated in EGFR (extracellular) followed by COX2 or COX1 followed by EGFR (intracellular) respectively. Quantification of signals was carried out with Quantity One software (Bio-Rad Laboratories AB, Sundbyberg, Sweden). One tumor sample was used as “standard sample” and loaded twice on each gel. The average optical density for the “standard sample” was used to normalize signal intensity between blots. Measured optical density is expressed as arbitrary units relative to the standard sample. MagicMark XP Western Protein Standards (Invitrogen) was used for molecular weight approximation.

### RNA extraction and cDNA synthesis

Total RNA from both tumor and mucosa samples from 30 patients was extracted with RNeasy® Fibrous Tissue Mini kit from Qiagen according to the protocol for Total RNA isolation from Fibrous Tissue enclosed by the manufacturer. A quality control measurement of RNA was performed in Bioanalyzer 2100 (Agilent) with limit RIN 5.0 for further analysis. Concentration of RNA was determined in NanoDrop® ND-1000 Spectrophotometer (NanoDrop Technologies). One μg of RNA was used in Advantage® RT-for-PCR kit (Clontech). Sterile water substituted for RNA in negative controls and samples without RT-polymerase were run to exclude genomic contamination.

### Q-PCR

Q-PCR was performed in the LightCycler 1.5 with LightCycler FastStart DNA Master^PLUS^ SYBR Green I kit to analyze the gene expression of COX-1, COX-2, and EGFR in tumor and mucosa tissue samples according to standard protocol (Roche) and with PCR conditions: activation 95°C 10 min, denaturation 95°C 10 s, annealing 60°C 4 s and extension 72°C 5 s in 45 cycles. Primers were Hs_PTGS1_1_SG (COX-1), Hs_PTGS2_1_SG (COX-2), and Hs_EGFR_1_SG (EGFR, detects all transcript variants) (QuantiTect Primer Assays, Qiagen). Two reference genes were run for each sample GAPDH (Hs_GAPDH_1_SG, Qiagen) and 18 S RNA (Hs_RRN18S_1_SG, Qiagen). Both reference genes were run with QuantiFast SYBR Green PCR kit (Qiagen) according to standard protocol and with PCR condition as follow: activation 95°C 5 min, denaturation 95°C 10 s and annealing 60°C 30 s in 40 cycles. The standard curve for COX-1 had a slope at-4.069, error 0.103 and R-0.99, COX-2 slope-3.674, error 0.07 and R-0.99, EGFR slope-3.896, error 0.076 and R-0.99, GAPDH slope-3.388, error 0.034 and R-1.00, and 18 S RNA slope-3.470, error 0.029 and R-1.00. All samples were run in duplicate and related to both reference genes ((GAPDH + 18SRNA)/2). The products were checked in the Bioanalyzer 2100 (Agilent Technologies) according to the protocol for DNA1000 for correct amplicon size. PCR-graded water was used as negative control in each reaction. Results were produced by the relative standard curve method where the standard specimen was a colon tumor (stadium III, RIN 7.3). All samples were diluted and confirmed to be within the range of the standard curve.

### Statistics

Results are presented as optical density (arbitrary units) for protein content or relative gene expression per GAPDH and 18 S RNA gene expressions as mean ± standard error of units obtained from the LightCycler® datafiles. The statistical testing among groups was performed with parametrical tests (ANOVA, Fisher PLSD) or Chi-square test (Microsoft Office Excel 2007). Correlation analysis was performed with either simple or multiple regressions according to standard procedures in Statview 5.0.1 (SAS Institue Inc.). P < 0.05 was regarded statistically significant and p < 0.10 a trend to significance in two-sided tests.

## Results

### Tissue protein content

**Figure 1:**
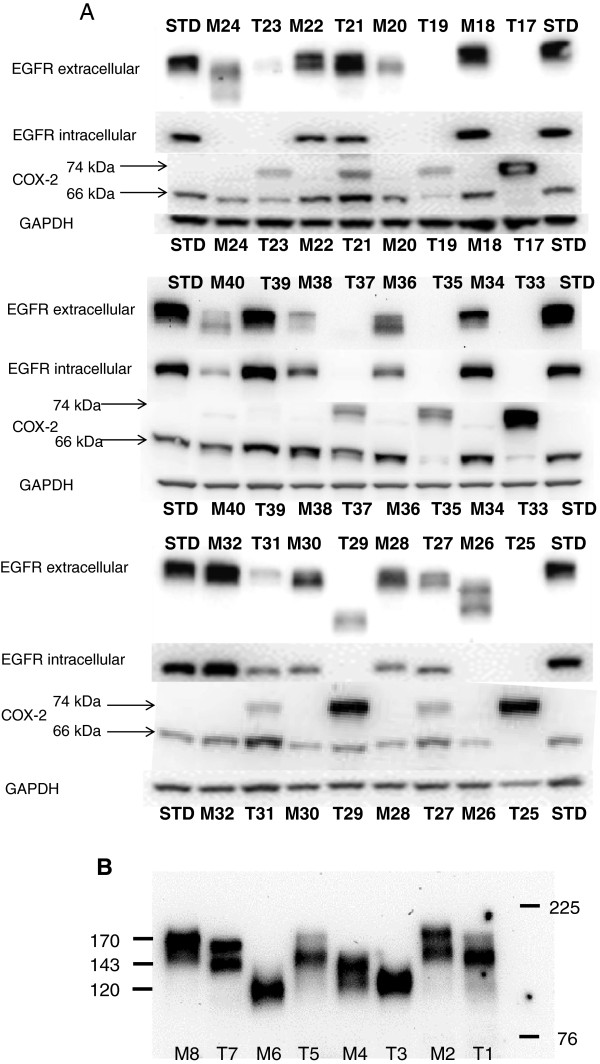
**Western blot gels for the two EGFR antibodies and the COX-2 antibody. A**. Two bands were detected with the COX-2 antibody, at approximately 66 kDa and 74 kDa. The antibody against the extracellular part of EGFR detected bands of different size. These bands are probably the different isoforms of EGFR. (STD = standard protein, M = mucosa, T = tumor). **B**. EGFR samples were separated in a Nupage Tris-Acetate gel for improved molecular weight separation. Molecular weight of proteins (kDa) was estimated from prestained molecular markers. Samples were randomly selected.

**Figure 2:**
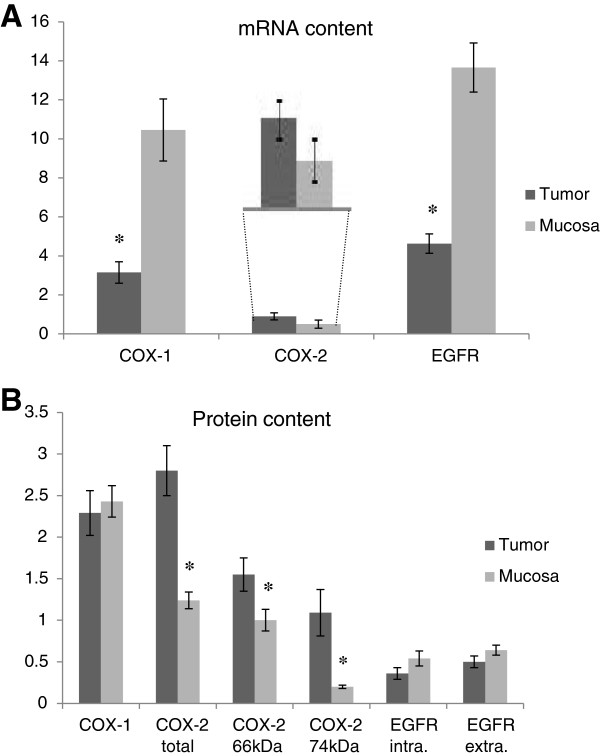
**mRNA and protein content in mucosa and tumor samples from the same patients.** (**A** = units/units (GAPDH + 18SRNA)/2, **B** = optical density, arbitrary units). *p < 0.05.

COX-1 protein was detected in all tumor and mucosa samples by western blot analyses except for one tumor tissue sample without significant difference between tumor and mucosa tissue (Table 1). The antibody against COX-2 detected two bands, one at ~ 66 kDa and one at 74 kDa. The band at 66 kDa was detected in almost all tumor and mucosa samples; only three tumors did not display the 66 kDa band. The band at 74 kDa was detected in 22 (73%) tumors and in 7 (23%) mucosa samples (Table 1). COX-2 protein content was significantly higher in tumor tissue compared to mucosa tissue for both 66 and 74 kDa (p < 0.03, p < 0.003, Table 2). Altered protein content in relationship to tumor progression was found for the 74 kDa band accounting for tumor stage (Table 2). The antibody against the intracellular part of EGFR detected only full length protein at 170 kDa in 19 (63%) tumors and in 22 (73%) mucosa samples. The antibody against the extracellular part of EGFR detected full length protein at 170 kDa in 20 (67%) tumors and 26 (87%) mucosa samples. Bands at lower molecular weight seemed to occur in samples with low or extinguished 170 kDa protein in tumor and mucosa tissue (Table 1, Figure 1A, B). EGFR protein was not significantly different between tumor and mucosa tissue (Figure 2).

**Table 1:** Number of patients with positive antibody detection in Western blot analysis of mucosa and tumor tissue

		**Mucosa (30)**	**Tumor (30)**	p*
COX-1		30 (100%)	29 (97%)	ns
COX-2	74 kDa	7 (23%)	22 (73%)	<0.0001
	66 kDa	30 (100%)	27 (90%)	ns
EGFR	Extracellular 170 kDa	22 (73%)	19 (63%)	0.01
	all sizes	30 (100%)	22 (73%)	ns
	Intracellular 170 kDa	22 (73%)	19 (63%)	0.01

*Chi-square test.

**Table 2:** COX-1, COX-2, and EGFR mRNAs and corresponding protein content in human colon mucosa compared to tumor tissue

		**Tumor tissue**				**ANOVA**	
	**Mucosa**	**Stadium I**	**Stadium II**	**Stadium III**	**Stadium IV**	**Tumor vs mucosa** **p value**	**Stage I-IV** **p value**
**RNA** (n)	(26)	(5)	(8)	(10)	(4)		
COX-1	10.45 ± 1.59	2.82 ± 0.90	3.40 ± 0.71	1.57 ± 0.34	7.04 ± 2.42	<0.0001	ns
COX-2	0.50 ± 0.21	0.27 ± 0.13	0.81 ± 0.21	1.02 ± 0.39	1.58 ± 0.68	ns	ns
EGFR	13.66 ± 1.26	4.90 ± 1.40	5.28 ± 0.89	3.37 ± 0.58	7.02 ± 0.83	<0.0001	ns
**Protein** (n)	(30)	(5)	(10)	(10)	(5)		
COX-1	2.43 ± 0.19	1.97 ± 0.50	1.92 ± 0.38	2.98 ± 0.61	1.97 ± 0.50	ns	ns
COX-2 total*	1.24 ± 0.10	1.53 ± 0.36	3.57 ± 0.68	2.34 ± 0.26	3.42 ± 0.70	<0.0001	I,II 0.002
							I,IV 0.009
COX-2 66 kDa	1.00 ± 0.13	1.25 ± 0.33	1.63 ± 0.51	1.59 ± 0.32	1.62 ± 0.22	0.028	ns
COX-2 74 kDa	0.20 ± 0.02	0.25 ± 0.09	1.70 ± 0.68	0.70 ± 0.22	1.49 ± 0.82	0.003	I,II 0.014
							II,III 0.036
EGFR extra	0.64 ± 0.06	0.91 ± 0.09	0.43 ± 0.12	0.40 ± 0.10	0.44 ± 0.15	ns	I,II 0.009
							I,III 0.006
							I,IV 0.023
EGFR intra	0.54 ± 0.09	0.40 ± 0.19	0.14 ± 0.06	0.54 ± 0.16	0.42 ± 0.15	ns	ns

Mean ± SEM, RNA: units/units ((GAPDH + 18SRNA)/2), protein arbitrary units. *COX-2 total=66 kDa + ~74 kDa protein band in WB. EGFR intra./EGFR extra. = ab directed towards intracellular/extracellular EGFR protein.

### Tissue mRNA content

**Figure 3:**
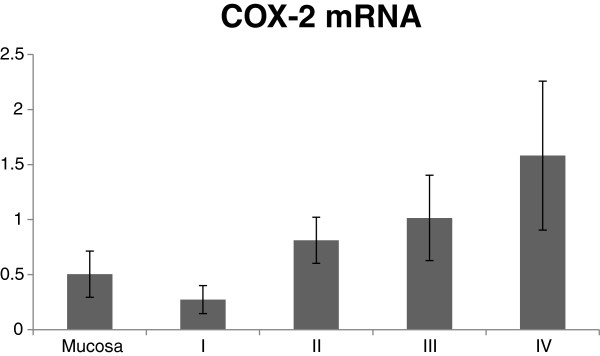
**Distribution of COX-2 mRNA content in tumor tissue related to tumor stage, which displayed a trend to increased expression with tumor progression.** (p < 0.04 in regression analysis, R 0.41).

**Figure 4:**
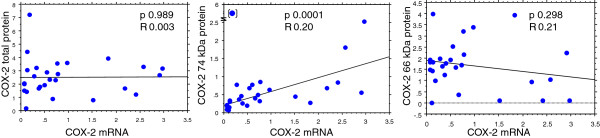
Regression analyses between COX-2 mRNA and COX-2 protein content in tumor tissue, which displayed a correlation for COX-2 mRNA and COX-2 74 kDa protein content.

Q-PCR displayed significantly reduced mRNA content in tumor tissue for COX-1 and EGFR (all transcripts), while COX-2 mRNA content was not significantly changed in tumor tissue compared to mucosa tissue (Table 2, Figure 2). COX-2 mRNA content in tumor tissue displayed a trend to increase with tumor progression (p < 0.04, Figure 3) where a weak correlation between COX-2 mRNA and the 74 kDa COX-2 protein content was observed in tumor tissue (p < 0.0001, Figure 4).

### COX-EGFR

**Figure 5:**
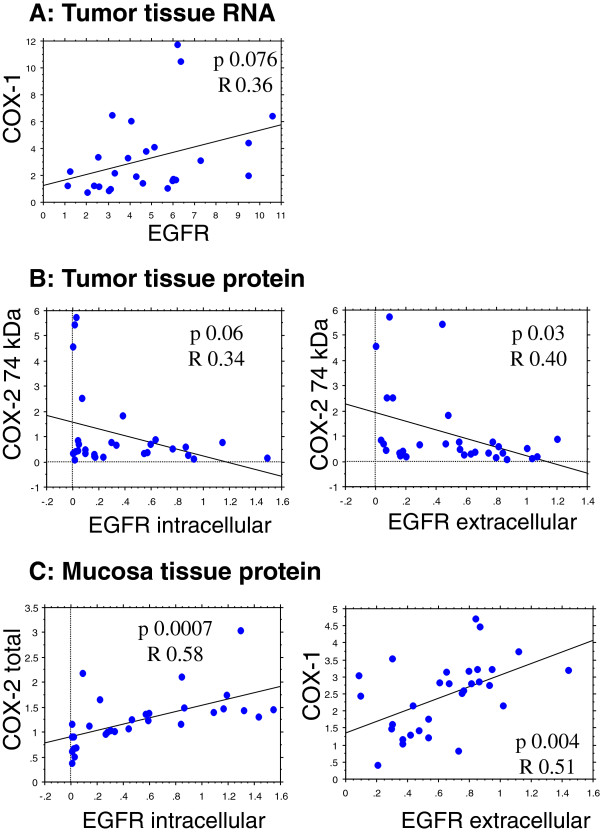
Statistically significant regression analyses of tumor and mucosa tissue: Panel A: RNA content (units/units reference genes) and panel B and C: protein content (arbitrary units).

Regression analysis between COX-1 mRNA and EGFR mRNA showed a trend to significance in tumor tissue (p < 0.07). Correlation analyses between either total COX-2 (~66 + 74 kDa) or the 74 kDa protein alone versus the intracellular and extracellular protein parts of EGFR in tumor and mucosa tissue displayed positive relationships between COX-1, total COX-2 and the extracellular or intracellular parts of EGFR in mucosa tissue (p < 0.06-0.0007). A negative correlation was seen between the 74 kDa COX-2 protein and the extra- and intracellular parts of EGFR in tumor tissue (Figure 5). Multiple regression analyses with intracellular EGFR protein as dependent variable displayed a correlation to the extracellular part of EGFR protein, total COX-2 and COX-1 protein as well as EGFR mRNA content in mucosa. No such relationships were observed in tumor tissue (Table 3).

**Table 3:** Multiple regression analyses with intracellular EGFR protein expression as dependent factor in tumor tissue (T) and mucosa tissue (M)

**Factor**	**Std. Coeff.**	**p-value**
EGFR extracell. protein		
M	1.11	<0.0001
T	0.61	0.0623
COX-2 total protein		
M	0.64	0.0006
T	-0.17	ns
EGFR RNA		
M	-0.02	0.0262
T	0.01	ns
COX-1 protein		
M	-0.29	0.0002
T	0.01	ns

Insignificantly independent variables were COX-1 RNA, COX-2 RNA, 66 kDa COX-2 protein and 74 kDa COX-2 protein.

## Discussion

Cyclooxygenases (COX) metabolize arachidonic acid to prostanoids, which are involved and control several important steps of tumor progression [5]. In normal human colon tissue there are two isoforms of COX; COX-1 is usually referred to as constitutively expressed in most tissues and COX-2 is induced by pathological conditions [4]. COX-1 has not been reported of in cancer to the same extent as COX-2 even though several reports indicate that COX-1 may be involved in tumor progression [15,16]. Also, our previous studies showed that tumor gene expression changed to less aggressive biological characteristics, as indicated by decreased cell proliferation, increased apoptosis and by more pronounced expression of immune markers in the tumors, following short term preoperative inhibition by indomethacin with a subsequent decline of both COX-1 and COX-2 [17,18]. Similar alterations are observed in different non-tumor related reactions of immunity, regulation of transcription and cell mobility following COX-inhibition.

Our results in the present study display that COX-1 mRNA was significantly higher in mucosa tissue compared to tumor tissue, which may indicate reduced production of COX-1 in tumor tissue. However, COX-1 protein contents were similar in tumor and mucosa tissue. The impact of reduced COX-1 mRNA in tumor tissue is unclear, but may be secondary to increased COX-2 protein and activity in tumor tissue.

Similarly, COX-2 mRNA was not significantly increased in tumor tissue versus mucosa tissue, which confirms our previous results [12], although there was a weak trend to increased COX-2 mRNA across tumor progression. By contrast, COX-2 protein content was significantly higher in tumor tissue in agreement with several previous reports [13,19]. COX-2 is tightly regulated and modified at several different levels in cell metabolism [20,21]. Post-transcriptional modifications like glycosylation are crucial for the activation and degradation of COX-2 [22]. COX-2 protein sequence contains 5 potential glycosylation sites where 3 are necessary for proper protein folding; one appears to affect the COX-2 activity, while one is usually not glycosylated. After glycosylation mature COX-2 is usually of 70-74 kDa size, while non-glycosylated COX-2 appears to be around 64 kDa following analythical electrophoresis [22-25]. Interestingly, non-glycosylated COX-2 protein (66 kDa) was detected in all tumor and mucosa samples, while mature COX-2 protein (74 kDa) was mainly detected in tumor tissue. The function of the 66 kDa COX-2 is unclear, but may represent unmature COX-2 that is to be either activated or remain inactive in the cell. These important observations with different molecular size patterns of variable COX-2 proteins in tumor and mucosa tissues may be highly significant but must await further analytical evaluations. Few publications have reported two bands of COX-2 in human tissues, while most reports comment on only one homogenous COX-2 protein (72-74 kDa) in animal tissue, although three distinct COX-2 bands have also been reported in monkey kidney cells [26]. Presently, it remains unclear too what extent two COX-2 bands are mainly biologically or methodology related.

Tumor tissue that contained increased mature and active COX-2 (74 kDa) protein seemed to lack epidermal growth factor receptor (EGFR) protein. In previous animal studies we found that EGFR and Kras mRNA were significantly decreased in tumor tissue from mice treated with unspecific COX inhibition [11]. Cross-talk between these two signalling pathways has been suggested by others where either COX-2 up-regulates EGFR or vice versa. Such results were mainly achieved in animal models or in cell culture experiments [8,9,27,28], where inhibition of both EGFR and COX in combination caused effective blockade of tumor growth and spread of metastatic disease in mice [8,29]. However, clinical trials have not displayed similar effects [10]. Accordingly, our present results in human tumor tissue displayed that increased COX-2 (74 kDa) was associated with low EGFR and vice versa. It is thus possible that COX-2 and EGFR signalling pathways are inversely related to each other in most colorectal tumors. Thus, it might be that only one pathway is highly active or that an alternative receptor to EGFR is present and activated, for example HER-2 signalling [30]. Our results are opposite to findings by others in a smaller group of patients [31,32]. On the other hand, a positive correlation between COX-1 and COX-2 (total protein content) versus EGFR protein content occurred in human mucosa tissue (Figure 5), although total COX-2 protein content (66 plus 74 kDa) may be less relevant in function, since non-glycosylated COX-2 (66 kDa) may lack enzymatic activity.

Human EGFR is encoded by two transcripts of 10.5 kb and 5.8 kb from a single promoter region/gene on chromosome 7. The protein products from these two transcripts are identical and encode the full-length receptor (isoform A, 170 kDa). In addition, three alternative transcripts of 2.4, 1.8 and 3.0 kb are derived from the *EGFR* gene. These transcripts encode isoforms B (unkown size), C (60/80 kDa), and D (90/110 kDa) respectively. All these isoforms lack the intracellular part of EGFR and therefore lack tyrosine kinase activity [33]. The electropheric bands we observed are probably isoforms of EGFR, which may compete for the ligand with full-length EGFR without giving rise to any internal signal.

EGFR plays a crucial role in cellular functions implicated in cancer development and is reported to be increased in tumor cells at tumor progression [34,35]. By contrast, our results displayed significantly decreased mRNA content of EGFR transcripts in tumor tissue compared to mucosa tissue without significant changes at the protein levels of EGFR. The explanation to this discrepancy is unclear, but an explanation may be observations that *K-RAS* mutations in tumor cells result in constant activity of EGFR signaling pathways, which might decrease EGFR mRNA by negative feedback due to cross-talk between EGFR activity and K-RAS function as observed in acquired resistance following anti-EGFR treatment of patients [36]. Thus, different alterations in turnover of mRNA and protein levels at either steady state or non-steady state conditions could display as divergent changes in cross-sectional evaluations on tissue samples. A second explanation may simply be that tumor EGFR mRNA was more susceptible to degradation by RNAse during tissue preparation compared to mucosa. In addition, tumor intestinal location may relate to different cell content of growth factors in tumors among right and left-sided CRC tumors [35]. The reason for a higher content of growth factors in left-sided tumors is unknown, but may be related to distributions of different cells along the large intestine. In our study 27% of the patients had left-sided tumors and 73% were right sided.

Overall, we found divergent alterations for mRNA and protein content of COX-2 in tumor tissue, which confirms our results in previous investigations [12]. These discrepancies may be due to the fact that mRNA molecules are comparatively unstable and may be more or less degraded during tissue handling [37]. Quantification of mRNA content in tissue may however be a valid means to measure increased or decreased alterations in protein production at specific conditions. Obviously, there are several critical events in measurements of mRNAs to define changes related to a specific receptor/enzyme level; also, post-transcriptional modifications may occur to various extent. The mRNA quality was rigorously checked in all present analyses and only RNA with RNA Integrity Number (RIN) above 5.0 were analyzed including two reference genes to overcome RNA quality hazards.

## Conclusion

In conclusion, the present study did not confirm correlations between tissue mRNA levels and protein content of COX-1 and EGFR, while a weak correlation was observed between the 74 kDa COX-2 protein and COX-2 mRNA in colon cancer tissue, accounting for tumor stage. By contrast, a negative correlation between COX-2 and EGFR protein in tumor tissue occurred, which was not observed in mucosa tissue from the same patients. Therefore, it is likely that COX-2 and EGFR signalling pathways are inversely related to each other in human colorectal tumor tissue and that tumor cells need only one of the signalling pathways for disease progression; a suggestion supported by our western blot results where tumor tissues with high COX-2 protein (74 kDa) content did not show EGFR protein expression at all. This fact should explain why combinatorial treatment with COX-inhibitors and anti-EGFR was not more effective than the single treatments alone [10].

## Abbreviations

COX-1/2: Cyclooxygenase-1/2; CRC: Colorectal cancer; EGFR: Epidermal growth factor receptor; RIN: RNA Integrity number.

## Competing interests

The authors declare that they have no competing interest.

## Authors’ contributions

AGA carried out the study design, gene expression studies, result analyses and drafted the manuscript. AF carried out protein analyses and gene expression studies. BMI participated and carried out protein analyses. HS carried out protein analyses. BG participated in study design and patient information. KL conceived of the study and participated in study design and helped drafted the manuscript. All authors have read and approved the manuscript.

## Pre-publication history

The pre-publication history for this paper can be accessed here:

http://www.biomedcentral.com/1471-2407/13/511/prepub
